# Free-running ultraviolet dual comb spectroscopy enabling absolute electronic fingerprinting

**DOI:** 10.1186/s43074-026-00250-6

**Published:** 2026-05-21

**Authors:** Lukas Fürst, Mithun Pal, Alexander Eber, Emily Hruska, Clemens Hofmann, Iouli E. Gordon, Martin Schultze, Rolf Breinbauer, Birgitta Bernhardt

**Affiliations:** 1 Institute of Experimental Physics, Graz University of Technology, Petersgasse 16, Graz, 8010 Austria; 2 Institute of Organic Chemistry, Graz University of Technology, Stremayrgasse 9, Graz, 8010 Austria; 3 Harvard-Smithsonian Center for Astrophysics, Atomic and Molecular Physics, 60 Garden Street, Cambridge, MA 02138 USA

**Keywords:** Ultraviolet Frequency Comb Generation, Dual-Comb Spectroscopy, High-Resolution Spectroscopy, Molecular Fingerprinting, Absolute Absorption Cross Sections, Formaldehyde

## Abstract

Ultraviolet (UV) spectroscopy reveals electronic transitions in matter that underpin atmospheric photochemistry and molecular dynamics. While dual-comb spectroscopy (DCS) has revolutionized precision measurements in the infrared region and expanded to the THz to the visible regions, its extension into the UV has been accomplished only very recently with demanding experimental efforts. Here we introduce free-running ultraviolet dual-comb spectroscopy (UV-DCS), a straightforward, high-fidelity method for absolute absorption cross sections and rapid molecular fingerprinting in the atmospheric UV window. The approach delivers high spectral resolution (1 GHz), broad bandwidth (12 THz), and fast acquisition (500 ms) without active stabilization, resulting in a UV-DCS quality factor that exceeds previously reported values.. Applied to formaldehyde (HCHO), a key atmospheric pollutant and photochemical driver, the technique yields an unprecedented count of rovibrational transitions in this window, enriching the molecular line list used for atmospheric chemistry and remote sensing. The measurements produce refined rotational constants, enabling high-accuracy quantum simulations of molecular eigenstates and informing ab initio models. Beyond robust absolute cross sections, UV-DCS provides a universal, rapid fingerprinting tool for transmissive species, offering fast atmospheric sensing and a rigorous benchmark for quantum theory without stabilization requirements. Coupled with advanced formaldehyde synthesis, these results support improved atmospheric monitoring, more reliable retrievals of HCHO abundances, and enhanced validation of fundamental molecular physics. Overall, this work realizes a free-running UV-DCS platform combining GHz resolution, multi-terahertz bandwidth, and sub-second acquisition, and applies it to refine formaldehyde ultraviolet spectroscopic parameters relevant to atmospheric and molecular science.

**Supplementary Information:**

The online version contains supplementary material available at 10.1186/s43074-026-00250-6.

## Introduction

Broadband and high resolution spectroscopy in the ultraviolet (UV) spectral region provides direct access to dense manifolds of electronic and vibronic transitions, where line positions and intensities encode detailed information on molecular electronic structure, vibronic couplings, and nonadiabatic dynamics such as dissociation or resonance interactions. High-resolution UV spectra, therefore, serve as stringent benchmarks for ab initio quantum-chemical calculations and enable the unraveling of excited-state pathways that are otherwise difficult to model.

One method offering simultaneously broadband coverage, high spectral resolution, and additionally rapid acquisition is dual comb spectroscopy (DCS) [1]. Only very recently, DCS has been expanded to the ultraviolet spectral region [2–6]. UV DCS has been proven to be highly effective for sensitive [2] and ultrabroadband [5] spectroscopic studies and for potential environmental applications [6].

UV DCS could become a pivotal method in atmospheric investigations because it aims at exploiting the strong electronic transitions whose absorption cross sections are orders of magnitude larger than those of rovibrational transitions in the infrared fingerprint region. This “electronic fingerprinting” makes UV DCS uniquely powerful for detecting trace concentrations of these species with high specificity. In this context, UV DCS represents a promising tool for air quality monitoring and for advancing our understanding of air pollution, which remains one of the foremost environmental challenges of the twenty-first century, contributing to more than five million deaths each year while adversely affecting human health, ecosystems, and climate change [7, 8].

Despite regulations that have resulted in substantial reduction of pollution in cities in the last decade, the health hazards remain. In response to the widespread and immediate consequences of air pollution, the European Council updated its ambient air quality directives in October 2024 to enforce stricter standards [9]. However, to effectively comply with the new regulations, it is essential to enhance our understanding of the earth's atmospheric condition. Both natural and anthropogenic activities influence its complex molecular composition, and with the sun driving photochemical reaction chains on multiple time-scales [10–13], it becomes necessary to monitor the fluctuating dynamics between interacting pollutant species.

Among all air pollutants, formaldehyde (HCHO) is a key volatile organic compound (VOC) that plays a pivotal role in atmospheric photochemistry [13–18] since it serves as a precursor to both hydroxyl radicals (HO_x_) and nitrogen oxides (NO_x_) and reacts to form secondary organic aerosols and tropospheric ozone (O_3_) [19, 20]. HCHO also holds a central role in the prebiotic chemistry of life’s key building blocks in the form of amino acids, nucleobases, and sugars; this motivates formaldehyde tracing in interstellar space [21]. Naturally elevated concentrations of HCHO are frequently detected in forested areas and regions susceptible to biomass burning, where it serves as an indicator of organic combustion processes [14]. However, anthropogenic activities, such as industrial emissions and vehicle exhaust, also act as sources for increased concentrations. Due to its toxicity and potential carcinogenic effects [22, 23], the air quality guidelines of the World Health Organization (WHO) establish a concentration limit of 0.1 mg/m3 to mitigate sensory irritation risks [24]. Furthermore, HCHO poses threats to ecosystems and biodiversity by negatively impacting plant health, diminishing crop yields, and harming aquatic organisms through both atmospheric deposition and industrial emission [25]. It is not a surprise that formaldehyde is one of the priority targets for multiple satellite and ground-based spectrometers operating in the ultraviolet (UV) [26]. This multifaceted impact underscores the importance of understanding, locating, and tracking formaldehyde emissions for public health, environmental management, and fundamental research.

In atmospheric chemistry, highly energetic UV light serves as a catalyst for an extensive array of photochemical reactions involving HCHO and other gaseous species (e.g., O_3_, NO_2_, and NO). In tropospheric ozone photochemistry, the reaction cascade following O_3_ photodissociation evolves via hydrogen radicals and HCHO, and their interaction with UV solar wavelengths between 340 and 365 nm constitutes a turning point of the reaction. When HCHO absorbs radiation below 365 nm, the cycle can terminate with a reduction of hydrogen radicals in ~2/3 of the reaction processes. Absorption of shorter wavelength radiation, however, evokes the production of methane and ammonia with a contrary effect of increasing the number of harmful hydrogen radicals HOx (~1/3 of the processes) [15].

Consequently, it is important to have reliable reference spectroscopic data for formaldehyde in this particular spectral region as they are one of the key input parameters for various atmospheric simulations [27] and fitting routines for satellite-based observations [26]. The HITRAN molecular spectroscopic database [28] provides line-by-line parameterization of formaldehyde only in the microwave and infrared spectral region, whereas for the UV part of the spectrum, only experimental cross-section can be used due to the difficulty in building a consistent and complete quantum mechanical model. Despite their importance, experimentally determined absorption cross sections known to date differ up to 20%, highlighting the challenge for absolute accuracy [17]. Probable origins of this uncertainty include the complexity of the potential reaction pathways with several branching reaction products, the tendency of formaldehyde to polymerize, and its affinity to surface adsorption. For the HITRAN database, Chance and Orphal [18] have recommended the use of cross-sections derived from the work of Ref [13]. with modifications (which included scaling of the intensities and introducing an offset) to achieve a better agreement with the lower resolution cross-sections from Ref. [16]. However, previous measurements of formaldehyde absorption cross sections have mainly been performed using grating-based spectrometers [16–18] and moving-mirror Fourier-transform spectroscopy [13, 15, 18]. These well-established approaches, generally require long acquisition times and offer spectral resolutions of several tens of gigahertz, which can be challenging for highly reactive species such as formaldehyde. To address challenges in obtaining reliable data experimentally, in addition to high resolution and sensitivity, extremely careful sample preparation and rapid acquisition times to minimize concentration changes during the measurement are requirements.

In this work, we introduce free-running UV dual comb spectroscopy (UV DCS), uniting all four of these prerequisites for the first time by employing passively-stable laser frequency combs without the need for complex cascaded frequency stabilization schemes. All DCS implementations in the UV so far require the long-term coherent superposition of two optical frequency combs. Due to the high frequency of UV radiation and the strong dispersion and absorption of all optical elements in this wavelength range, achieving mutual coherence requires extensive active stabilization schemes or sophisticated feed-forward techniques [1, 29]. This is achieved by using two combs that share a single cavity, thereby experiencing nearly identical dispersion, nonlinear phase accumulation, and environmental perturbations. As a result, strong common-mode noise suppression and intrinsically high mutual coherence are obtained, which are maintained even after frequency up-conversion to the UV spectral region. Here, we have developed an ultraviolet free-running dual-comb spectrometer for the first time without the need for active feedback, allowing the measurement of absorption spectra with 1 GHz frequency comb resolution in less than a second acquisition time.

A detailed comparison of this system with existing UV dual-comb spectroscopy platforms is provided in Supplementary Table S1, highlighting key metrics such as signal-to-noise ratio, acquisition speed, spectral resolution, optical bandwidth, and dual-comb quality factor. Our detection scheme achieves a factor of 20 reduction in noise, together with enhanced spectral resolution and sensitivity for HCHO spectroscopy, compared to the best previously reported FTS-based measurements in this ultraviolet spectral range [15]. Resulting from this advance, our method resolves almost one order of magnitude more rovibrational transition energies than have so far been experimentally observed [18]. These rovibronic transitions have been theoretically predicted but, so far, evaded conclusive experimental observation due to their narrow linewidths and low transition strength.

To provide theory-driven studies with absolute, rather than relative, transition probabilities for the refinement of empirical simulations and the understanding of atmospheric photochemistry, extremely particular sample conditions must be met. First, the knowledge of the exact number density in the measured sample is required. Additionally, the presence of other molecular contaminants, which may ultimately contribute their own spectral features to the absorption signal or offer novel reaction pathways, must be minimized. Here, we ensure these specifications are met by preparing pure HCHO in the gas phase via an robust double-distillation synthesis scheme. Both the instrumental development and its application to HCHO spectroscopy illustrate the two-fold innovation of this work in both ultraviolet dual-comb methodology and high-resolution molecular characterization.

## Experimental scheme and methods

### Experimental setup

**Fig. 1:**
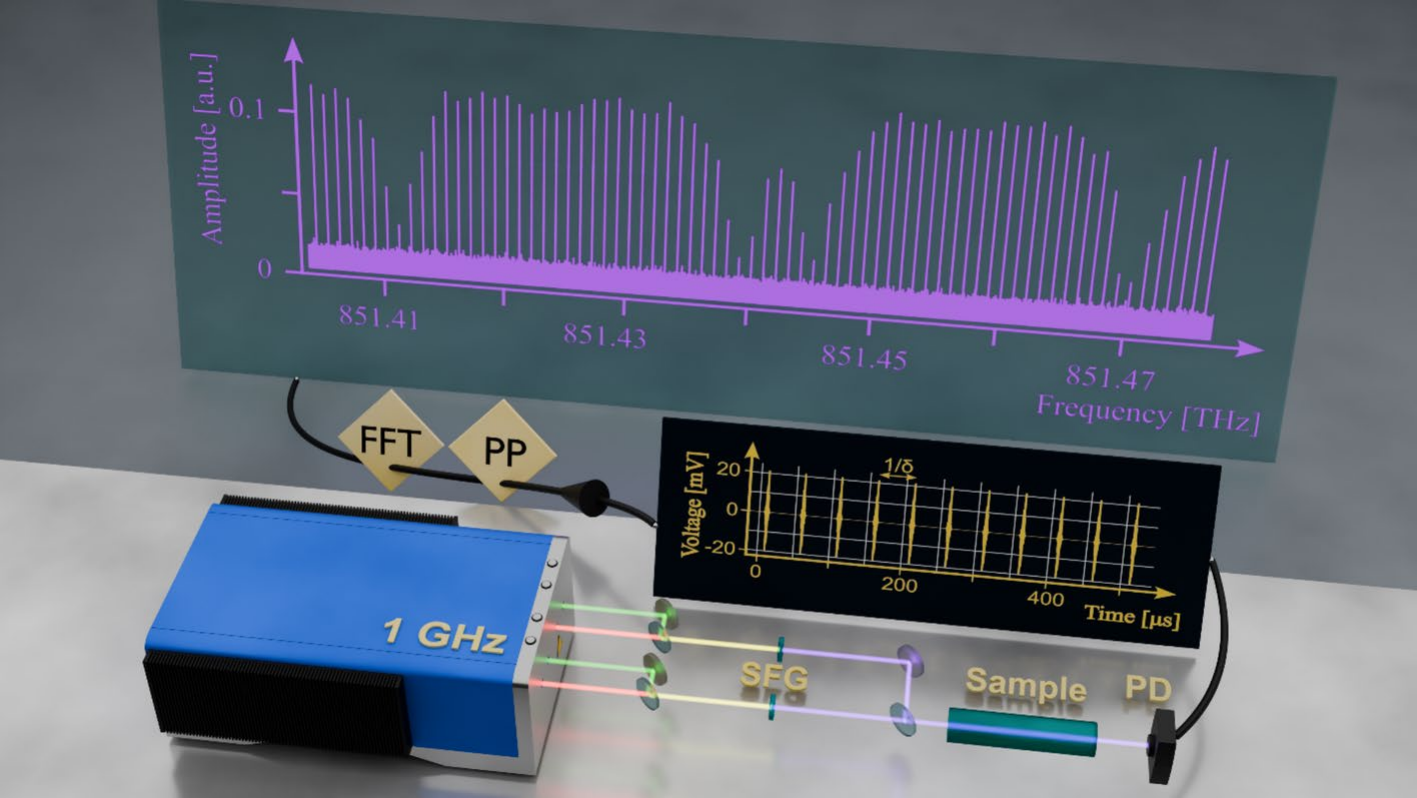
Experimental scheme of single-cavity dual comb spectroscopy in the ultraviolet. The ultraviolet frequency combs are generated via frequency up-conversion of infrared combs using sum-frequency generation (SFG) between the fundamental laser and the second harmonic. Both UV beams pass through the sample cell containing purified HCHO and generate a time-dependent interference signal on a fast photodiode (PD, see black screen). The time trace is post-processed (PP) using a phase self-correction algorithm. The fast Fourier transformation (FFT) yields transmission spectra with comb-mode resolution (see gray screen)

Figure 1 depicts the experimental scheme of free-running UV DCS. The laser source used in this work is a commercially available Yb:CALGO crystal-based, single-cavity dual-comb oscillator operating in the near-infrared. It generates two optical frequency combs with center frequencies of approximately 285.7 THz (wavelength 1050 nm) and 285.0 THz (wavelength 1052 nm). The average output powers of the two combs are 2.5 W and 2.1 W, respectively, with pulse durations of approximately 100 fs. Mode-locking is achieved using a semiconductor saturable absorber mirror (SESAM) in a diode-pumped solid-state laser configuration [30]. The two optical frequency combs from a single laser cavity are spatially multiplexed using a Brewster-angled biprism. The laser system operates in free-running mode without active stabilization of the comb parameters. We did not observe any time limit for coherent averaging. The repetition rates measured are *f*_rep,1_ = 1 GHz and *f*_rep,2_ = 1 GHz + 19 kHz. For each of the two infrared combs, a waveplate and a polarizing beamsplitter send a variable fraction of the fundamental radiation to a nonlinear crystal for second harmonic generation (SHG). This process yields two additional frequency combs in the visible spectral region centered at 525 ± 10 nm and 526 ± 10 nm, respectivley. For comb 1 and 2 the maximum average power of the visible outputs is 350 mW and 310 mW, respectively, with pulse durations of approximately 90 fs. In order to generate the near-UV dual-comb outputs, the fundamental (IR) and second-harmonic (VIS) beams are spatially overlapped using a dichroic mirror. An off-axis parabolic mirror tightly focuses the beams into a beta barium borate (BBO) crystal with a thickness of 0.8 mm to facilitate SFG. A delay stage is used to ensure precise temporal overlap between the IR and VIS pulses for efficient SFG. After optimization, 170 mW of VIS and 920 mW of IR power were found to maximize the UV output radiation to 5.5 mW. The UV frequency comb had a central wavelength of 350.5 nm, corresponding to an optical frequency of 855.4 THz. Notably, the system exhibited excellent long-term stability: no measurable decrease in UV output power or degradation of beam quality was observed over more than one month of continuous operation. Moreover, beam pointing remained highly stable throughout measurements, underscoring the mechanical and optical robustness of the system. These characteristics highlight the reliability and practicality of this approach for implementing a compact, turn-key UV dual-comb spectrometer suitable for extended laboratory and field applications. Then the two ultraviolet beams are spatially superposed at a plate beamsplitter, and the interferogram is detected using a fast photodiode.

The repetition rate difference $\delta$ results into two pulse trains with a sweeping delay between consecutive pulses that causes an interference burst every $1/\delta$= 1/19 kHz = 53 µs, called an interferogram. The optical delay is analogous to the varying pathlength difference in Fourier-transform spectroscopy using a scanning-mirror interferometer, resulting in a down-converted heterodyne signal. The detuning of 19 kHz ensures alias-free RF mapping while preserving the full optical bandwidth in a single acquisition.

An oscilloscope card reads the signal from the photodiode and streams the data of the interferogram chain to storage. A phase-correction algorithm applied post-recording compensates for residual beating frequency drifts due to slow noise components originating from mechanical resonances. Detailed information on the post-processing procedure is provided in the Supplementary Information. While post-processing effectively compensates slow phase drifts [31, 32], it does not mitigate fast noise components, such as relative intensity noise (RIN), which contribute to the system not reaching the shot-noise limit [33]. Nevertheless, the observed linear scaling of SNR with optical power indicates that detector noise is the dominant noise source in the present measurements.

After Fourier transformation of the interferogram, the obtained radio frequency spectrum can be up-converted to the UV domain by multiplying the frequency axis with the down-conversion factor [1, 30] $m={f}_{rep}/\delta \sim 5\times {10}^{4}$. With coherent averaging of consecutive interferograms under stable comb conditions, a high signal-to-noise ratio (SNR) is achieved. Simultaneously, through time multiplexing, the required optical power is reduced [1].

For HCHO spectroscopy measurement, the overlapped beams pass through the HCHO sample cell.

For the determination of the absolute absorption cross section, HCHO is prepared in an advanced two-step purification process to provide precise knowledge about the sample concentration with calibrated pressure gauges (see Supplementary). All acquisitions were performed with a duration of 0.5 s. This short acquisition time was chosen to minimize the influence of effects such as wall adsorption and photodissociation. Additionally, the 0.5 s short measurement offers an optimal balance between signal-to-noise ratio (SNR) and the temporal resolution required for trace gas analysis.

Besides the sample cell, the whole beam path is in air. We define the baseline for absorption measurements using a baseline fitting routine. Assuming linearity of the Beer-Lambert law, we calculate the absorption cross section values using the HCHO pressure, the length of the absorption cell, and laboratory temperature. We use a gas-type independent capacitance pressure gauge and a purified, liquid reservoir of monomeric HCHO (see section preparation of monomeric formaldehyde).

### Sample preparation

Monomeric formaldehyde is prepared in a two-step purification process. The resulting liquid is transferred to a stainless-steel reservoir and connected to the vacuum setup. The whole vacuum setup is heated and evacuated overnight to warrant low contamination, e.g. from water. The sample cell is evacuated to the 10^–2^ mbar range. Then, we let gas-phase HCHO expand into vacuum until the desired pressure is reached, which is monitored by a calibrated, gas-type-independent capacitance pressure gauge. The pressure gauge is calibrated, i.e. zero-adjusted, at a background pressure of less than 10^–6^ mbar and has an accuracy of 0.2% of the measured value. The optical path through the sample cell has a length of (62.0 ± 0.3) cm with windows oriented at Brewster’s angle. For the results presented, a multi-pass geometry with 5 passes was used, which yields a total interaction length of (310.0 ± 1.5) cm. The pressure is monitored during measurements for precise determination of the HCHO concentration.

## Results

### Free-running ultraviolet dual-comb spectroscopy

**Fig. 2:**
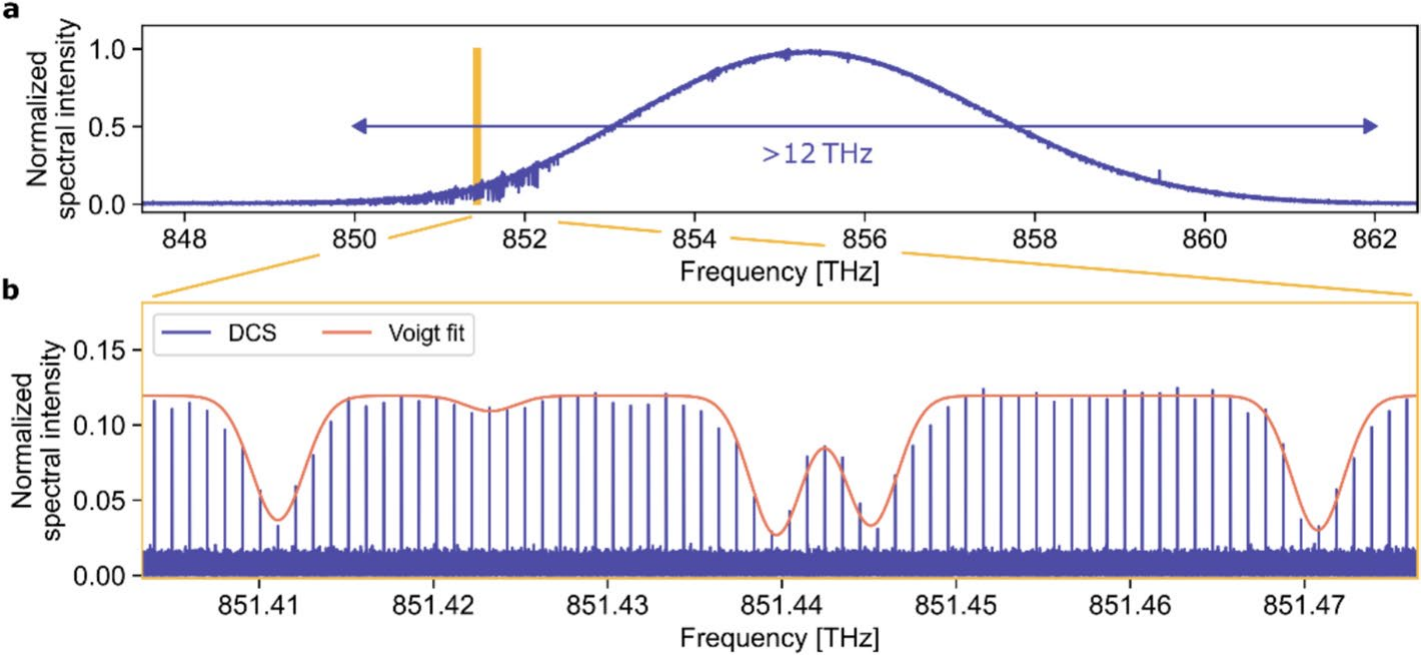
Broadband near-ultraviolet dual-comb spectrum. **a** The near-ultraviolet, coherently-averaged spectrum is centered at 855.4 THz and covers a full bandwidth of 12.4 THz, resolving the congested absorption features between 850 THz and 852.5 THz and around 855 THz. Here, 9,650 interferograms were coherently averaged over a single interferogram period (52.6 µs), yielding the spectral envelope. **b** Comb-mode-resolved absorption spectrum of the phase-corrected signal using a 500 ms-long trace, fully resolving the comb structure with 1 GHz mode spacing. Eventually, the absorption dips of HCHO are sampled with a spectral resolution of 1 GHz

Figure 2a displays the mean spectrum obtained after coherent averaging of 9,650 single interferogram bursts. Both frequency combs possess a high mutual coherence with a low relative optical frequency noise, enabling detection of the UV spectrum with a SNR of 255 within 500 ms acquisition time. The SNR is determined as the inverse of the standard deviation of the normalized transmission spectrum evaluated in spectral regions free of absorption features (see Supplementary). The spectrum extends over a bandwidth of ~12 THz including more than 10^4^ individually-resolved comb modes and covering more than 600 rovibronic absorption lines of HCHO. The resulting dual-comb quality factor, defined in [34] is $QF\text{ = SNR }\times \frac{\Delta f}{\delta f \cdot \sqrt{T}}= \text{255 } \times \frac{\textrm{12.4THz}}{\text{1 GHz }\cdot \sqrt{\textrm{0.5 s}}}\approx \textrm{ 4.5 }\times {10}^{6}\sqrt{\textrm{Hz}}$, with a spectral resolving power of ~10^6^, the highest reported QF value to date. Figure 2b shows the comb-mode-resolved spectrum with five characteristic HCHO absorption features. The frequency comb modes have transform-limited linewidth and are spaced by 1 GHz, corresponding to the repetition rate of the laser.

### Broadband absorption spectroscopy of formaldehyde

**Fig. 3:**
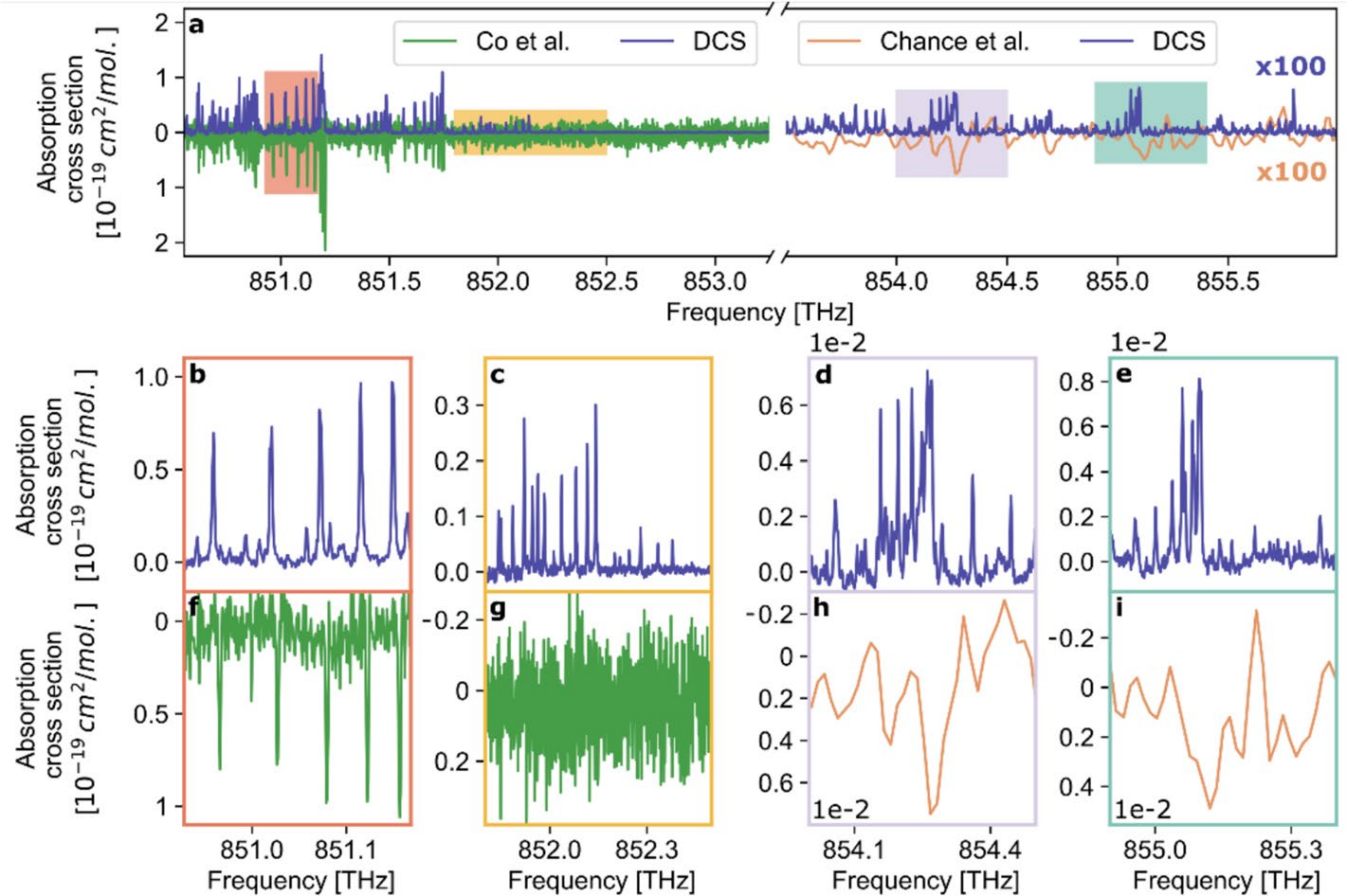
Comparison of ultraviolet absorption cross section data. **a** DCS absorption cross section spectrum of HCHO (blue, above the abscissa) compared to previous measurements [15, 18]. The spectrum was recorded at T = 294 K, p(HCHO) = 90 mbar, with an optical path length of 310 cm and an acquisition time of 0.5 s. **b** & **f** The strong absorption features of both traces agree qualitatively but display minor differences in the line positions. **c** & **g** The DCS spectrum has an up to 20 times lower noise level, despite a substantially shorter acquisition time (0.5 s for DCS versus 60 min in Ref. [15]). **d**, **e** & **h**, **i** For frequencies above 853 THz, the DCS trace resolves the rovibronic lines for the first time. The mean relative absorption cross section uncertainty amounts to 7.2%

To benchmark our results, Fig. 3 compares the UV DCS absorption spectrum to state-of-the-art measurements with the highest reported resolution in this spectral window [15, 18]. The linewidths of the absorption features measured in this work agree with previous studies and the predicted width assuming Doppler broadening (T = 294 K, ν_FWHM_ = 1905.5 MHz). We observe a minor relative frequency offset on the order of 10^–6^ in the line positions between the DCS measurement and the literature (see Fig. 3b & f). The frequency axis was calibrated using a two-step procedure. First, the RF frequency axis was mapped onto the optical frequency axis by applying the measured values of the comb repetition rate and the repetition-rate detuning. The frequency offset was determined by comparing the simulated absorption spectrum produced with the previously-reported transition frequency of the 4^1^_0_ vibronic band (848.79 THz ± 150 MHz) [35, 36] to the measured DCS spectrum. The absolute accuracy of the frequency scale is therefore limited by the uncertainty of the calibration reference of ±150 MHz. Due to the high SNR of 255, DCS permits the detection of previously unresolved weak absorption lines down to ${10}^{-22 }{\mathrm{cm}}^{2}\mathrm{/mol}$, which are observed at frequencies above 851.8 THz (see Fig. 3c-e & g-i, note the different y-scale). The rotational linewidths and positions of these lines are investigated for the first time because of an improvement in SNR and spectral resolution compared to state-of-the-art of more than one order of magnitude. The uncertainty of the absorption cross-section measurement is determined to be 7.2% based on repeated measurements performed under identical conditions. For each repetition, the absorption cross section is calculated, and the standard deviation across repetitions is evaluated for each absorption peak. The reported value represents the mean of these standard deviations over all detected peaks, thereby accounting for instrumental fluctuations, including residual amplitude fluctuations and phase noise in the time-domain interferograms affecting the baseline stability of the free-running dual-comb system, uncertainties in the pressure gauges, and variations in signal detection.

### Determination of the absolute absorption cross section of formaldehyde

To substantiate the recorded absorption spectrum with theory, we perform simulations of the absorption cross section spectrum of HCHO using PGOPHER [37] with the rotational parameters, including centrifugal distortion constants, from Ref. [35, 38]. The simulation enables assigning the corresponding transitions, including all relevant quantum numbers. We then carried out a new fit using our data. Knowledge about the line positions and intensities allows for precise testing of rotational constants and investigation of perturbations and higher-order corrections [39].

**Fig. 4:**
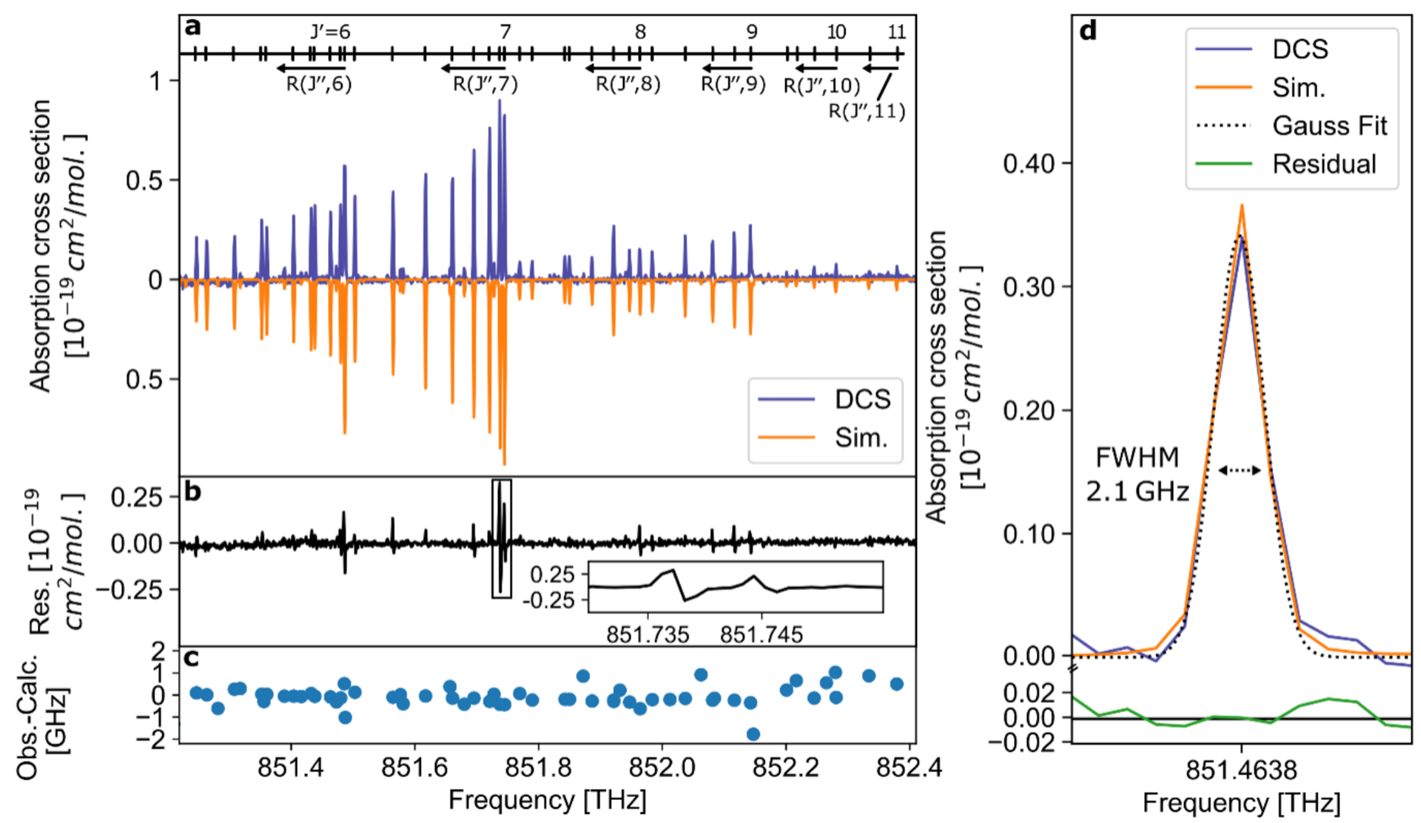
Comparison between simulation and dual comb spectroscopy data. **a** DCS absorption cross section of HCHO (4^1^_0_-vibronic branch, T = 294 K, 310 cm pathlength, 1.07 mbar pressure, 0.5 s acquisition time) in comparison to a simulated spectrum (Sim.) [37]. **b** The residual values (Res.), i.e., the difference between the DCS and simulation curve, reveal minor differences in the absorption cross section of less than 0.25 × 10^–19^ cm^2^/mol. **c** The obtained line positions show very good agreement with a standard deviation of 0.37 GHz. **d** A single absorption line centered at 851.404 THz and the corresponding Gaussian fit. The full-width-at-half-maximum (FWHM) amounts to 2.1 ± 0.1 GHz (1σ standard deviation), consistent with the expected width assuming Doppler broadening and within the 3σ uncertainty range

Figure 4 shows the comparison between the simulation using our new constants and the experimental DCS spectrum. The two spectra are in excellent agreement, as evidenced by low residual values (see Fig. 4b). The line positions agree with the simulation with a standard deviation of 0.37 GHz, reflecting the precision of the fit and remaining below the spectral resolution (see Fig. 4c). The short-term precision of f_rep_​ was evaluated from the RMS fluctuation provided by the laser control unit, yielding a standard deviation of approximately 130 Hz over a 5-min interval. For a repetition rate of ~1 GHz, this corresponds to a relative stability of ~1.3 × 10^−7^. Fluctuations of Δf_rep_ are mitigated through the applied phase-correction algorithm prior to spectral reconstruction, confirming that the frequency-scale precision does not limit the observed line-position agreement.The full-width-at-half-maxima of the Gasussian line fits are in excellent agreement with the linewidth expected from Doppler broadening (T = 294 K), as the example in Fig. 4d shows.

**Fig. 5:**
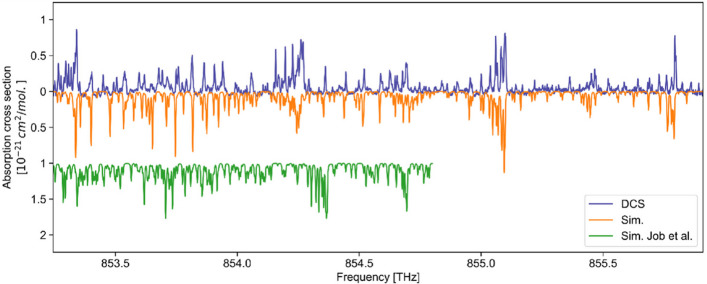
High-resolution rovibronic absorption spectrum of formaldehyde. **a** Measured absorption cross section spectrum of HCHO (DCS, T = 294 K, 310 cm pathlength, 90 mbar pressure, 0.5 s measurement time) including rotational states that have not been experimentally observed to date. The measured absorption lines do not obey periodic branching like in the 4^1^_0_ band and have an absorption cross section on the order of ${10}^{-22 }{\mathrm{cm}}^{2}\mathrm{/mol}$. The simulation (Sim.) is performed with our improved simulation parameters compared to the literature, fitting to the experiment (see Supplementary) [37, 40]. The green trace (Sim. Job et al.) shows the simulation based on the previously reported parameters from Job et al. [39], included here for direct comparison. The absence of prior high-resolution experimental data above 854.8 THz highlights the novelty of the present measurements

Figure 5 displays the absolute absorption cross section of HCHO between 853.2 THz and 856 THz. We detect a complex structure of rotationally-resolved absorption features, characteristic for ultraviolet absorption spectra where electronic, vibrational, and rotational degrees of freedom are involved, which makes an unambiguous assignment challenging. This absorption spectrum constitutes the first experimental resolution of rotational transitions in HCHO (see Fig. 3h) in this spectral range. The corresponding simulation and measurement data are provided in the supplementary material. We assign the absorption lines to the weak 3^1^_0_4^2^_1_ vibronic branch, and the simulation is in good agreement with the measured DCS trace using improved rotational constants A, B, C, and centrifugal distortion terms for this branch, helping to refine quantum mechanical models (see Supplementary) [40]. These analysis efforts extend the knowledge about the electronic fingerprint and molecular structure of HCHO in the important ultraviolet region further. These results also show our absolute absorption cross-section data compared with simulations using the currently listed constants [40], illustrating the significant improvement obtained with the updated parameters. Moreover, the presented spectral window extends beyond that covered in Job et al. [40], representing a previously unexplored region both experimentally and in simulation.

## Discussion

Atmospheric processes include dissociation channels for frequencies above 833 THz (λ < 360 nm wavelength) via excited electronic states and photochemical or photophysical oxidation, where knowledge about the absolute absorption cross section is indespensable [41–43]. Our work adds significantly to the knowledge of the formaldehyde behavior in this photochemically-active spectral region that plays an important part in crucial tropospheric pollution monitoring projects like TEMPO, TROPOMI and GEMS [10, 11, 26]. Although the present system operates in a fully free-running regime, referencing the comb repetition rate to an external RF standard would improve frequency-axis stability further, and absolute frequency accuracy could be achieved with additional optical referencing, for example via beat-note detection of the fundamental comb with a stabilized continuous-wave (CW) laser. Our main focus is that the compact and robust design of the system presented in this work makes it well-suited for mobile environmental sensing applications, usually not requiring frequency comb precision.. Most notably, DCS real-time monitoring is now possible with unprecedented temporal resolution after the most recent development of streaming self-corrected dual comb spectroscopy [44]. Based on our previous works with similar setups [44] and considering our recently achieved long path field geometries of 5 km, we estimate the HCHO detection limit of the presented system to 2.5 ppb for an averaging time of 150 s and an interaction pathlength of 5 km, typical for atmospheric sensing. The detailed calculation can be found in the supplementary information. Given the standard tropospheric abundance of formaldehyde between 1–4 ppb, implementation of the presented system in an open-path configuration with a retroreflector, analogous to the arrangement demonstrated by Eber et al. [44], would enable atmospheric field measurements under realistic outdoor conditions. Our new system does not require additional optical stabilization schemes and can be operated without extensive electronic supplies. In terms of simplicity, only Xu et al. [2] could report on a simplified, EOM-based UV dual-comb spectrometer that achieves comb-resolved spectra. We realize a spectral bandwidth of more than 12 THz in a single measurement, spanning across numerous absorption features of HCHO. Investigating HCHO directly in the atmosphere would yield valuable information about its complex photochemistry and pre-dissociation, especially in areas where precarious concentrations are found, e.g. industrial areas and biomass burnings [14, 43, 45]

Enhancing the spectral bandwidth to frequencies above 884 THz (339 nm wavelength), where high absorption cross sections of up to ${{4}\times {10}}^{-19 }{\mathrm{cm}}^{2}\mathrm{/mol}$ are observed, paves the way towards ultra-sensitive detection of formaldehyde even at the ppt-level. This becomes achievable with direct spectral broadening in the UV [6] or by spectral broadening in the IR and subsequent SFG with optimized phase matching for the targeted UV region. Furthermore, literature values of the absorption cross section in the extended photochemically active spectral region still differ significantly up to now [41, 46]. An alternative detection method with high sensitivity and unprecedented resolution, as demonstr ated here, will help resolve this spectroscopic conundrum, which is decisively relevant for environmental monitoring at these very low levels.

The differences in absolute line intensities between measurement and simulation can be explained by perturbations such as Coriolis-type interactions between different vibrational modes and shifts in line-center positions. Deviations in line-center positions contribute significantly to the residuals in Fig. 4b, especially for point symmetric features where line-position mismatch dominates. Asymmetric residuals are instead explained by resonance interactions between nearby molecular states, which can alter transition probabilities by up to 200% [39] and are not accounted for in current simulations. Through the calibration of the absorption line intensity, our absolute absorption cross section data warrants a precise analysis that helps toward the understanding of processes such as pre-dissociation [41].

In comparison to state-of-the-art methodology in high-resolution spectroscopy in the UV, which includes synchrotron laboratories, tunable frequency up-converted laser spectroscopy, and Fourier-transform spectroscopy, our method provides a compact, cost-effective, and rigid measurement concept with record short acquisition times and unparalleled spectral resolution. This renders UV dual-comb spectroscopy suitable to decisively advance environmental sensing applications [47], (laboratory) astrophysics [48], and medical diagnosis [49].

## Conclusions

In conclusion, we have, for the first time, demonstrated free-running, broadband ultraviolet dual-comb spectroscopy that achieving the highest quality factor reported to date in this spectral region. The UV generation scheme is implemented in a straightforward and robust architecture**,** enabling a true turn-key UV frequency comb platform that can be readily utilized. This spectrometer resolves hundreds of previously undetected rovibrational transitions of formaldehyde and provides rotationally resolved absolute absorption cross-section spectra that yield a precise electronic fingerprint of formaldehyde as test sample with huge environmental impact. The absorption line positions were measured with a relative accuracy better than 10⁻⁶, estimated as approximately half the effective spectral resolution divided by the optical carrier frequency ($\frac{{\Delta \vartheta }_{res}/2}{{\vartheta}_{0}}$). For frequencies beyond 853 THz, the system delivers an order-of-magnitude improvement in spectral resolution over state-of-the-art techniques. This breakthrough establishes UV-DCS as a powerful and accessible tool for precision molecular spectroscopy, bridging a critical gap in the ultraviolet domain. Its combination of high resolution (1 GHz)**,** broad bandwidth (12 THz), and instrumental simplicity (free-running, i.e. without any stabilization requirements) will establish it as a transformative platform for real-time atmospheric monitoring**,** benchmarking of quantum molecular simulations**,** and next-generation photochemical research. Beyond formaldehyde, this approach opens the door to high-fidelity UV spectroscopy of a wide range of environmentally and chemically relevant species (e.g. O_3_, HONO and NO_2_) and can be seamlessly adopted by laboratories worldwide.

## Supplementary Information

Supplementary Material 1.

## Data Availability

The data that support the findings of this study are available from the corresponding authors upon request.

## Abbreviations

DCS: Dual-comb spectroscopy
UV: Ultraviolet
VOC: Volatile organic compound
WHO: World Health Organization
SHG: Second harmonic generation
SNR: Signal-to-noise ratio
PD: Photodiode
PP: Post-processed
FFT: Fast Fourier transformation
FWHM: Full-width-at-half-maximum
BBO: Beta barium borate
SFG: Sum-frequency generation
HCHO: Formaldehyde
TEMPO: Tropospheric Emissions Monitoring of Pollution
GEMS: Geostationary Environment Monitoring Spectrometer
EOM: Electro-Optic Modulator
HITRAN: High-Resolution Transmission Molecular Absorption Database
RF: Radio Frequency
TROPOMI: Tropospheric Monitoring Instrument
