# Supplementary material for: Free-running ultraviolet dual comb spectroscopy enabling absolute electronic fingerprinting
Source: Photonix. 2026 May 21;7(1):33. doi: 10.1186/s43074-026-00250-6 (PMC13194232; doi:10.1186/s43074-026-00250-6)
Supplement: Supplementary file 1 — Supplementary Material 1. [file 43074_2026_250_MOESM1_ESM.docx]

Supplementary Information

Free-running Ultraviolet Dual Comb Spectroscopy enabling Absolute Electronic Fingerprinting

Lukas Fürst^1^, Mithun Pal^1^, Alexander Eber^1^, Emily Hruska^1^, Clemens Hofmann^2^, Iouli Gordon^3^, Martin Schultze^1^, Rolf Breinbauer^2^, Birgitta Bernhardt^1^*

*^1^Institute of Experimental Physics, Graz University of Technology, Petersgasse 16, 8010 Graz, Austria*

*^2^Institute of Organic Chemistry, Graz University of Technology, Stremayrgasse 9, 8010 Graz, Austria*

*^3^Harvard-Smithsonian Center for Astrophysics, Atomic and Molecular Physics, 60 Garden Street, Cambridge, MA, 02138, USA*

*Correspondence author: bernhardt@tugraz.at

### **Supplementary Note 1: Comparison of free-running UV-DCS with existing UV-DCS implementations**

### As summarized in Supplementary Table S1, prior systems typically exhibit trade-offs between SNR, bandwidth, and acquisition times. Optimizing all measurement parameters at the same time is challenging and for some even impossible. The dual comb quality factor[S1] $QF=SNR\times\frac{\Delta f}{\delta f\cdot\sqrt{t}}$ shows the correlation of different parameters and enables a useful opportunity to compare different DCS realizations. For instance, while Muraviev et al.[S2] (QF = 3.6×10^6^ *√Hz*) demonstrated a large optical bandwidth (74 THz) and high SNR (588), this was achieved over an exceptionally long acquisition time exceeding 6 hours (23,220 s). Similarly, Xu et al.[S3] (QF = 1.8×10^6^ √*Hz*) demonstrated high SNR values (260) with moderate resolution (>200 MHz), but required acquisition times ranging from 64 to 328s. They achieved QF ranging from 1.6×10^6^ to 2.6×10^6^ *√Hz*. Other systems, such as that of McCauley et al.[S4], achieved shorter acquisition times (1.1s) but with significantly lower QF (0.13 × 10⁶ √Hz).

### In contrast, the free-running UV DCS system presented here delivers a SNR of 255 within only 0.5s, i.e. real time, over a broad spectral bandwidth of 12.4 THz and with 1 GHz resolution—yielding a QF of 4.5 × 10⁶ √Hz, the highest among all systems compared. Importantly, these results were obtained without the use of active phase stabilization or feed-forward correction schemes, highlighting the simplicity of the presented experimental scheme for future implementation.

# **Supp. Table ST1: Comparative performance metrics of UV dual-comb spectroscopy systems with SNR being signal to noise ratio, t acquisition time, QF dual comb quality factor defined as** $\boldsymbol{QF}\mathbf{=}\boldsymbol{SNR}\boldsymbol{\times}\frac{\boldsymbol{\Delta f}}{\boldsymbol{\delta f}\boldsymbol{\cdot}\sqrt{\boldsymbol{t}}}$

| **Reference** | **Center**  **Wavelength**  **(nm)** | **SNR** | **Acquisition time t  [s]** | **Resolution** $\boldsymbol{\delta f}$ **[MHz]** | **Bandwidth** $\boldsymbol{\Delta f}$**[THz]** | **QF  [√Hz]** |
| --- | --- | --- | --- | --- | --- | --- |
| Xu  et al.[S3] | 389 | 195 | 152 | 500 | 0.05 | 1.6×10^6^ |
| Xu  et al.[S3] | 387.7 | 210 | 64 | 500 | 0.05 | 2.6×10^6^ |
| Xu  et al.[S3] | 388.97 | 260 | 328 | 200 | 0.026 | 1.8×10^6^ |
| Chang et al.[S5] | 386 | 757 | 100 | 100 | 3 | 2.3×10^6^ |
| McCauley et al.[S4] | 265 | 117 | 1.1 | 1200 | 1.4 | 0.13×10^6^ |
| Muraviev et al.[S2] | 391 & 333.5 | 588 | 23,220 | 80 | 74 | 3.6×10^6^ |
| Fürst et al.[S6] | 344 | 23.4 | 8 | 50,000 | 35.7 | 5.9×10^3^ |
| This work | 352.1 | 255 | 0.5 | 1000 | 12.4 | 4.5×10^6^ |

### **Supplementary Note 2: Post-processing phase correction algorithm**

### The post-processing methodology used in this work closely follows the approach described in Refs[S7, S8]. In brief, the time-domain interferograms acquired by the free-running dual-comb spectrometer are corrected using a self-referenced phase correction algorithm that compensates for residual temporal phase fluctuations between the two combs. This correction is crucial for enabling coherent averaging of successive interferograms, which in turn significantly enhances the signal-to-noise ratio (SNR). Importantly, the algorithm preserves the intrinsic frequency accuracy of the comb lines, thereby allowing the SNR to scale proportionally with the square root of the total acquisition time.

### The algorithm operates by tracking and correcting temporal variations in the carrier-envelope phase of the interferogram train. This is achieved through the use of cross-ambiguity functions, which estimate instantaneous frequency fluctuations and phase drifts. The extracted phase error is subsequently used to realign the interferograms in the time domain, ensuring coherent summation during averaging.

### **Supplementary Note 3: Preparation of monomeric formaldehyde**

### HCHO is an irritant chemical, which is toxic by skin contact and inhalation, and is a known carcinogen. Therefore, its preparation and disposal have to be carried out in a well-ventilated fume hood wearing personal protective equipment (lab coat, safety glasses, nitrile protective gloves).

### This setup was adapted from previous works[S9–S11], but instead of a specialized apparatus commercially-available glass equipment employed in synthetic chemistry laboratories is used. The cracking and distillation process is performed in a single apparatus.

### The experimental setup for the cracking of paraformaldehyde involves a glass apparatus composed of a Schlenk flask (see Fig. S1, left) connected by a glass bridge to a cooling trap (see Fig. S1, middle), which is again connected via a glass bridge to a receiving Schlenk flask (see Fig. S1, right). The glass apparatus is connected to a Schlenk-type argon/vacuum manifold.

### Before performing the cracking process, about 5 g paraformaldehyde (extra pure, Merck, K12297505) is dried in a NS29 Schlenk flask by heating in an oil bath at 80 °C under vacuum (~1 mbar) for 16 h followed by 2 h at 100 °C.

### The glass apparatus depicted in Figure S1 is evacuated to < 0.5 mbar and dried thoroughly using a heat gun. After ventilation with argon the Schlenk flask containing the dried paraformaldehyde is connected (see Fig. S1, left) and the glass apparatus again evacuated and heated thoroughly.

### After the apparatus cools to room temperature under vacuum, the first cold trap is cooled in a Dewar with liquid nitrogen. The Schlenk flask containing the paraformaldehyde is immersed in an oil bath (see Fig. S1), which is gradually heated to 150 °C. This process should be performed carefully in order to minimize paraformaldehyde particles, which might start “jumping” in the course of the depolymerization process due to gas evolution. During the entire cracking process, the oil pump vacuum holds the receiving Schlenk flask at ~ 0.6 mbar. The monomeric formaldehyde, as well as residual water, collects in the upper region of the intermediary cold trap as a white solid.

### After a sufficient amount of paraformaldehyde has depolymerized, the oil bath is removed and the cryogenic distillation of the monomeric formaldehyde is pursued. The receiving Schlenk flask is cooled in a Dewar filled with liquid nitrogen (-196 °C) and the liquid nitrogen Dewar of the intermediary cooling trap is subsequently replaced with a Dewar filled with dry ice/acetone (-78 °C).

### The connection to the vacuum pump is closed. The solidified formaldehyde in the cooling trap melts as it warms to the temperature of the dry ice/acetone bath and distills into the receiving Schlenk flask. In order to maintain vacuum in the apparatus, every 5-10 min the connection to the vacuum pump is opened shortly. After a sufficient amount of formaldehyde has been distilled, it is transferred via a cannula into a specially-designed stainless-steel reservoir possessing a septum and an integrated hose-adapter.

### Preparing for the transfer, the stainless-steel vessel is connected to a second Schlenk line via the integrated hose adapter and rendered inert by heating under vacuum with a heat gun. Then, continuous argon flow is applied. A cannula, which has been freshly taken from a drying oven at 120 °C, is plunged through the septum, and the lower collection compartment is cooled to -78 °C in an dry ice/acetone bath. Argon flow is maintained through the cannula up until the cannula transfer of the liquid.

### In parallel, the glass apparatus is flushed with argon through the connection to the receiving Schlenk flask. While maintaining constant argon flow, the receiving flask is disconnected from the apparatus, closed with a rubber septum and immediately immersed into a Dewar filled with dry ice/acetone (-78 °C). At this temperature, the distilled formaldehyde forms a colorless liquid. In order to avoid the release of formaldehyde vapors from the unused part of the cracking and distillation glass apparatus, a dummy flask is used to close off the glass apparatus again.

### The liquid from the receiving Schlenk flask is transferred to the stainless-steel vessel by plunging the other end of the cannula through the septum of the Schlenk flask. By applying overpressure of argon in the Schlenk flask and providing slight pulses of vacuum to the stainless-steel vessel, the transfer succeeds within seconds. The stainless-steel vessel is disconnected from the Schlenk line and transported in a dry ice/acetone Dewar to the laser apparatus. It remains in this Dewar during the entire period of the measurements.

### Disposal of formaldehyde

### Any remaining formaldehyde in the receiving flask and the stainless-steel vessel is carefully quenched by the addition of distilled water under argon at -78 °C. A bubbler containing 0.1 M NaOH is attached before the flasks are left to warm up to room temperature overnight. The used glassware pieces are thoroughly cleaned from repolymerized paraformaldehyde with 0.1 M NaOH, an NaOH/iPrOH base bath (24 h), and a 0.1 M HCl bath (24 h).


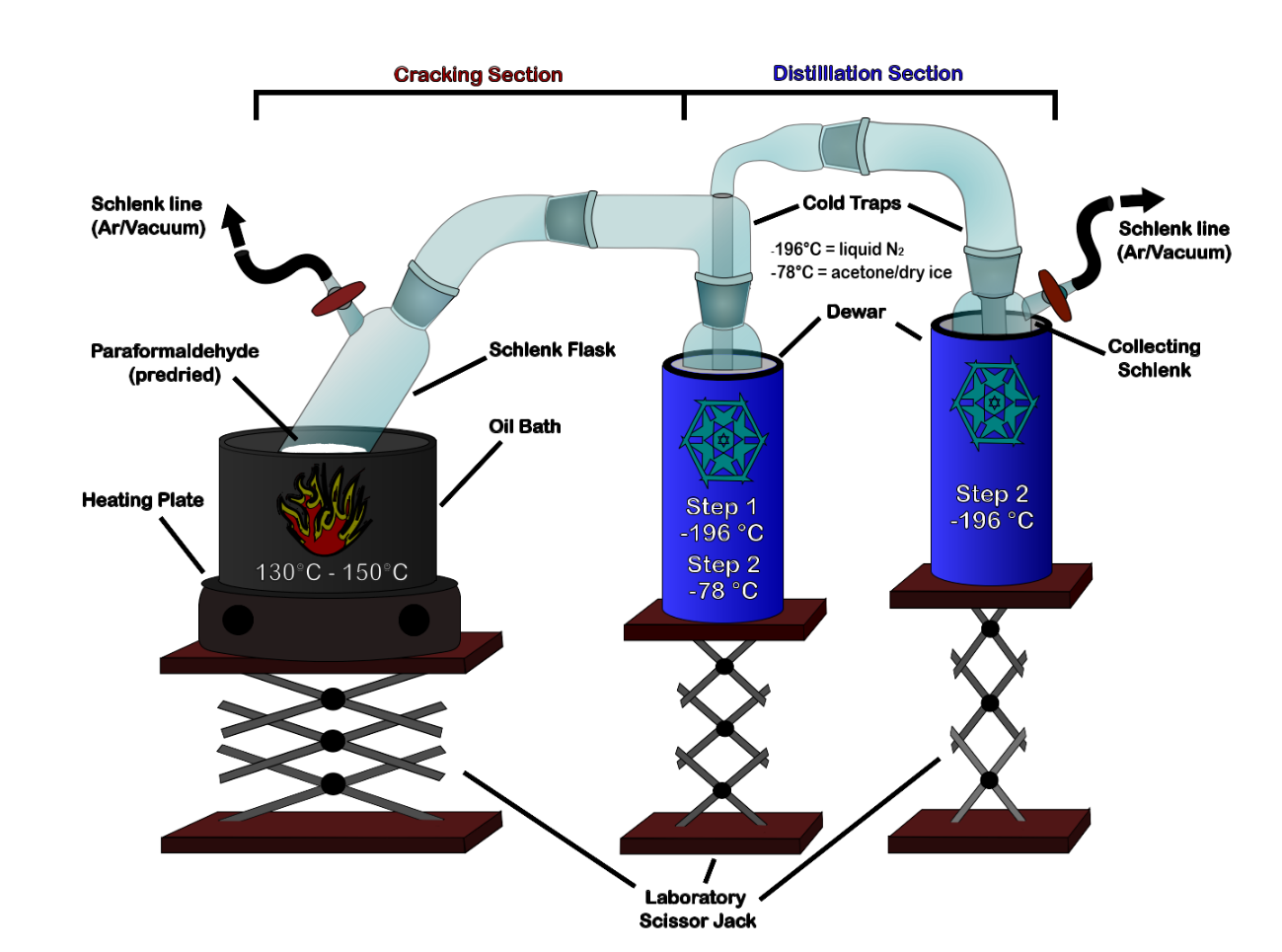


**Figure S1.** Experimental apparatus for the preparation of monomeric formaldehyde.

Formaldehyde is collected in the cooling trap in the middle, which is cooled to -196°C using liquid nitrogen during the cracking process (Step 1). For the purification process (Step 2) the formaldehyde from the cooling trap is distilled into the receiving Schlenk flask, which is cooled in a Dewar to -196°C.**Potential Impurities**

There are two main potential impurities, water (H_2_O) and 1,3,5-trioxane. To exclude contributions of these species to the absorption spectra, control measurements using pure water vapor and pure 1,3,5-trioxane have been performed under comparable conditions, confirming that neither exhibits detectable absorption features within the investigated ultraviolet spectral window. The effectiveness of the purification is ensured by the staged cryogenic trapping, employing a dry ice-acetone bath to freeze out contaminants like H_2_O and 1,3,5,-trioxane. Controlled distillation and temperature control of the traps during transfer further suppress impurity carryover.

### **Supplementary Note 4: Absorption cross-section calculation**

The absolute absorption cross section (Eq. 1) is calculated from the absorption (A) measured using the UV-DCS system, based on the Beer–Lambert law. The absolute absorption cross section $\sigma$ (in cm²/molecule) is given by:

$$\sigma=A\times\ln\left( 10 \right)\times\frac{K_{B}T}{p\times l}, A=-\log_{10}\left( \frac{I_{sample}}{I_{reference}} \right) 1$$

where k_B_ =1.38064852×10^-23^ J/K is the Boltzmann constant, T is the absolute temperature in Kelvin, p is the pressure in pascals, and l is the optical path length in meters.

For the determination of absolute absorption cross sections, we carefully ensured that the stronger HCHO absorption features (particularly those near 850 THz) were measured under partial pressure (0.1 mbar) conditions, resulting in maximum absorption below 5% of the spectral intensity, well within the linear regime of the Beer-Lambert law. To detect weaker transitions, the concentration of HCHO in the measurement cell was increased accordingly; however, under these conditions, the stronger lines became saturated and were ignored.

**Supplementary Note 5: Free-running dual-comb characterization**

### We characterize our dual-comb spectrometer by investigating the scaling of the signal-to-noise-ratio (SNR) versus measurement time and average power on the photodiode. The spectral SNR is calculated as the inverse of the standard deviation of the normalized transmission spectrum from spectral regions exhibiting no absorption features, similar to previous work[S1]. Figure S2 illustrates the SNR variation over a 2 THz spectral window where there is no absorption. Figure S2a shows that the SNR scales with the square root of measurement time. The SNR increases for higher average powers on the detector with a linear dependence (see Fig. S2b). However, the SNR scaling in Fig. S2 confirms that the measurement is not shot-noise limited, which can be attributed to the dominance of technical noise sources, primarily detector noise from the built-in transimpedance amplifier and laser RIN. The photodetector also exhibits saturation above 5 mW optical power, likely due to early amplifier saturation, which reduces dynamic range and introduces nonlinearities and excess noise. These effects collectively limit SNR improvement, even with increased optical power. A potential improvement would be to replace the current detector with a fast avalanche photodiode (APD), which offers higher saturation thresholds and lower noise, thereby mitigating the detector-noise limitation, albeit at a significantly higher cost.


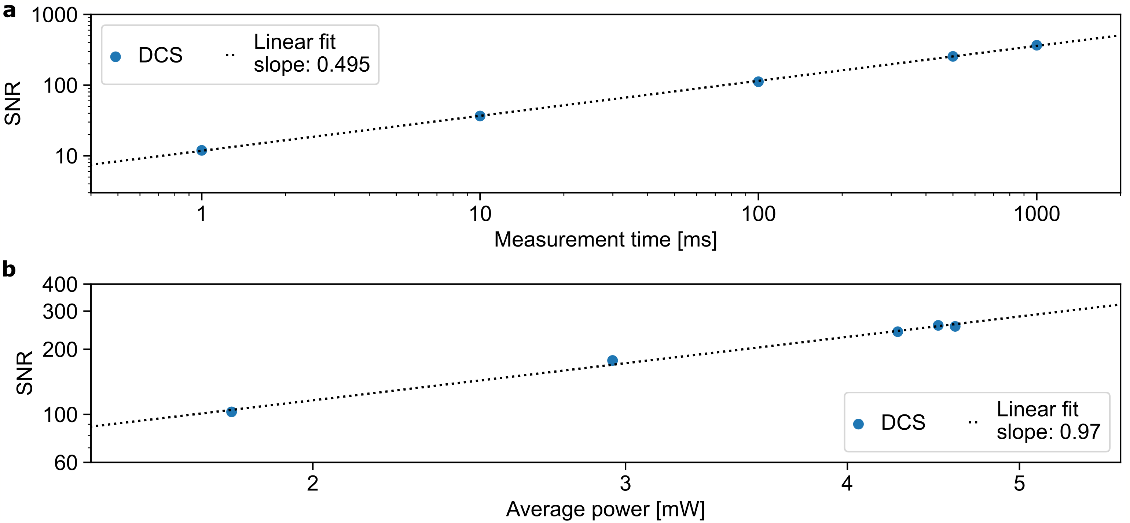


**Figure S2. Signal-to-noise-ratio (SNR) of free-running dual comb spectroscopy. (a)** The SNR is plotted versus measurement time and follows a square-root scaling as expected. **(b)** The SNR increases for higher average powers at the detector.

**Supplementary Note 6: Calculation of limit of detection (LOD)**

Following standard absorption-spectroscopy practice, we define the limit of detection as the concentration corresponding to SNR = 1. We provide an experimentally grounded estimate by scaling the measured absorption-to-noise ratio. Assuming linear absorbance scaling with pressure and path length, the detection limit (LOD) can be expressed as

$$LOD\left( ppb \right)= {\sigma_{noise}}^{-1}\times\frac{p_{meas}}{p_{atm}}\times\frac{L_{meas}}{L_{new}}$$

Here σ_noise is 1σ baseline absorption noise, p_meas is the measurement pressure, p_atm is atmospheric pressure, L_meas is the interaction path length during the laboratory measurement, and L_new is the assumed atmospheric open-path length

Using the experimentally determined absorption-to-noise ratio of 20, a measurement pressure of 1bar influencing the line shape of the absorption feature, a laboratory path length of 3.1 m, and an assumed atmospheric open-path length of 5 km, we obtain LOD ≈ 32 ppb for the acquisition time of 0.5 s.

# Further improvements in sensitivity are expected through increased interaction path length and/or longer acquisition times for ambient concentration measurements. Since the limit of detection scales inversely with the square root of the averaging time (LOD∝ 1/√t ) [S12], extending the acquisition time from 0.5 s to 150 s would reduce the detection limit to approximately 2.5 ppb.

Supplementary Tables

Table ST2.

Simulation constants of the 4^1^_0_ branch (see Fig. 4) and the 3^1^_0_4^2^_1_ branch (see Fig. 5).

| Vibronic branch | 4^1^_0_ | 3^1^_0_4^2^_1_ |
| --- | --- | --- |
| Origin [MHz] | 848789500 ± 90 | 8534100100 ± 3800 |
| A [MHz] | 262376 ± 4 | 259450 ± 251 |
| B [MHz] | 33720 ± 4 | 44660 ± 160 |
| C [MHz] | 30325 ± 4 | 15860 ± 170 |
| DK [MHz] | 15.01 ± 0.03 | -2.9 ± 9 |
| DJK [MHz] | 1.960 ± 0.012 | -11 ± 3 |
| DJ [MHz] | 0.065 ± 0.003 | -16.6 ± 0.7 |
| $\boldsymbol{\Delta}$K [MHz] | -3.3 ± 0.7 | 316 ± 7 |
| $\boldsymbol{\Delta}$J [MHz] | 0.066 ± 0.003 | 46.4 ± 0.3 |

Supplementary Files

Data S1. “S1_hcho_294K_310421.pgo” (separate file)

PGOPHER file containing the simulation with the adjusted rotational parameters.

Data S2. “S2_hcho_294K_410.pgo” (separate file)

PGOPHER file containing the simulation with the adjusted rotational parameters.

Data S3. “S3_hcho_294K_310421_852.5-858.5THz(1GHz_DCS).txt” (separate file)

TXT file containing the measured data of the 3^1^_0_4^2^_1_ band.

Data S4. “S4_hcho_294K_410_848.931-857.779THz(1GHz_DCS).txt” (separate file)

TXT file containing the measured data of the 4^1^_0_ band.

Data S5. “S5_hcho_294K_410_linelist.txt” (separate file)

TXT file containing the absorption line list of the measured data of the 4^1^_0_ band.

Supplementary References

S1. Newbury NR, Coddington I, Swann W (2010) Sensitivity of coherent dual-comb spectroscopy. Opt Express 18:7929

S2. Muraviev A, Konnov D, Vasilyev S, Vodopyanov KL (2024) Dual-frequency-comb UV spectroscopy with one million resolved comb lines. Optica 11:1486–1489

S3. Xu B, Chen Z, Hänsch TW, Picqué N (2024) Near-ultraviolet photon-counting dual-comb spectroscopy. Nature 627:289–294

S4. McCauley JJ, others (2024) Dual-comb spectroscopy in the deep ultraviolet. Optica 11:460

S5. Chang KF, others (2024) Multi-harmonic near-infrared–ultraviolet dual-comb spectrometer. Opt Lett 49:1684–1687

S6. Fürst L, others (2024) Broadband near-ultraviolet dual comb spectroscopy. Optica 11:471

S7. Hébert NB, others (2017) Self-corrected chip-based dual-comb spectrometer. Opt Express 25:8168–8179

S8. Hébert NB (2024) SelfCorrectIGMs (Retrieved October 25, 2024)

S9. Walker F (1933) Some Properties of Anhydrous Formaldehyde. J Am Chem Soc 55:2821–2826. https://doi.org/10.1021/ja01334a030

S10. Smith GD, Molina LT, Molina MJ (2002) Measurement of radical quantum yields from formaldehyde photolysis between 269 and 339 nm. Journal of Physical Chemistry A 106:1233–1240. https://doi.org/10.1021/jp013180n

S11. Spence R, Wild W (1935) The preparation of liquid monomeric formaldehyde. J Chem Soc 338–340

S12. Eber A, Gruber C, Schultze M, et al (2025) Streaming self-corrected dual-comb spectrometer. Opt Express 33:35314–35325
